# Post-Mortem Biomarkers in Sudden Cardiac Death: From Classical Biochemistry to Molecular Autopsy and Multi-Omics Forensic Approaches

**DOI:** 10.3390/ijms27020670

**Published:** 2026-01-09

**Authors:** Matteo Antonio Sacco, Helenia Mastrangelo, Giuseppe Neri, Isabella Aquila

**Affiliations:** 1 Institute of Legal Medicine, Department of Medical and Surgical Sciences, “Magna Graecia” University, 88100 Catanzaro, Italy; matteosacco@unicz.it (M.A.S.); heleniamastrangelo@gmail.com (H.M.); 2 Anesthesia and Intensive Care, Department of Medical and Surgical Sciences, “Magna Graecia” University, 88100 Catanzaro, Italy; giuseppeneri91@gmail.com

**Keywords:** sudden cardiac death, molecular autopsy, biomarkers, multi-omics

## Abstract

Sudden cardiac death (SCD) remains a major challenge in forensic medicine, representing a leading cause of natural mortality and frequently occurring in individuals without antecedent symptoms. Although conventional autopsy and histology remain the cornerstones of investigation, up to 10–15% of cases are classified as “autopsy-negative sudden unexplained death,” underscoring the need for complementary diagnostic tools. In recent years, post-mortem biochemistry and molecular approaches have become essential to narrowing this gap. Classical protein markers of myocardial necrosis (cardiac troponins, CK-MB, H-FABP, GPBB) continue to play a fundamental role, though their interpretation is influenced by post-mortem interval and sampling site. Peptide biomarkers reflecting hemodynamic stress (BNP, NT-proBNP, copeptin, sST2) offer additional insight into cardiac dysfunction and ischemic burden, while inflammatory and immunohistochemical markers (CRP, IL-6, fibronectin, desmin, C5b-9, S100A1) assist in detecting early ischemia and myocarditis when routine histology is inconclusive. Beyond these traditional markers, molecular signatures—including cardiac-specific microRNAs, exosomal RNA, proteomic alterations, and metabolomic fingerprints—provide innovative perspectives on metabolic collapse and arrhythmic mechanisms. Molecular autopsy through next-generation sequencing has further expanded diagnostic capability by identifying pathogenic variants associated with channelopathies and cardiomyopathies, enabling both cause-of-death clarification and cascade screening in families. Emerging multi-omics and artificial intelligence frameworks promise to integrate these heterogeneous data into standardized and robust interpretive models. Pre- and post-analytical considerations, together with medico-legal implications ranging from malpractice evaluation to the management of genetic information, remain essential components of this evolving field. Overall, the incorporation of validated biomarkers into harmonized international protocols, increasingly supported by AI, represents the next frontier in forensic cardiology.

## 1. Introduction

Sudden cardiac death (SCD) is a leading cause of natural mortality, accounting for approximately 15–20% of all natural deaths and an estimated 4–5 million fatalities per year worldwide [1]. It is defined as an unexpected natural death of presumed cardiac origin occurring within one hour of symptom onset, or within 24 h of the individual last being seen alive in good health if unwitnessed [2]. The causes of SCD vary with age: coronary artery disease predominates in adults over 35 years, whereas younger victims more frequently present cardiomyopathies, myocarditis, or inherited arrhythmic syndromes [3,4]. From a forensic perspective, the investigation of SCD poses unique challenges. Despite the central role of autopsy and histopathology, 10–15% of cases remain unexplained after complete post-mortem examination and are classified as “autopsy-negative sudden unexplained deaths” (SUDs) [5,6,7,8,9,10,11,12,13,14,15,16,17,18].

Recent epidemiological assessments from international registries and cardiovascular societies confirm that SCD constitutes a substantial proportion of natural mortality across different regions, with age-specific incidence and underlying mechanisms varying significantly among populations [19]. These observations have prompted major bodies, including the World Health Organization and the European Society of Cardiology, to emphasize structured diagnostic pathways that integrate clinical, imaging, and molecular data, underscoring the need for equally robust and standardized approaches in the post-mortem setting. Such cases pose particular challenges in the medico-legal context, leaving families without definitive explanations, courts without conclusive causal evidence, and clinicians without essential data to guide prevention. Basso et al. reported that unexplained sudden deaths in the young often reveal molecular or electrical substrates invisible to routine histology, reinforcing the importance of complementary diagnostic approaches [6].

In this regard, post-mortem biomarkers have emerged as valuable tools to address the diagnostic gap. Protein markers of myocardial injury, such as cardiac troponins and CK-MB, have been extensively studied, with evidence supporting their ability to differentiate ischemic cardiac deaths from non-cardiac causes [7]. Peptidic biomarkers, including BNP, NT-proBNP, copeptin, and soluble ST2, reflect hemodynamic stress and acute neurohormonal activation, providing additional discriminatory value [8]. Inflammatory and immunohistochemical markers such as CRP, IL-6, fibronectin, desmin, complement C5b-9, and S100A1 contribute to the identification of early ischemia and myocarditis, particularly when routine histology is inconclusive [9,10].

Recent research has further expanded into the molecular domain. MicroRNAs (miRNAs) demonstrate high post-mortem stability and are highly specific for cardiomyocyte injury. Multiple studies have shown that cardiac miR-1, miR-133, miR-208, and miR-499 are elevated in sudden ischemic deaths and often correlate with troponin levels [11]. Exosomal miRNAs may exhibit even greater resistance to degradation, enabling improved discrimination between ischemic and arrhythmic mechanisms [12]. In parallel, proteomic and metabolomic profiling have revealed specific metabolic patterns associated with ischemic collapse, although these signatures require careful interpretation in relation to post-mortem interval [13]. Cao et al. demonstrated that integrating metabolomic fingerprints with protein biomarkers enhances classification accuracy in unexplained SCD, particularly when supported by machine-learning algorithms [14].

A major advance in the field is the development of molecular autopsy. Next-generation sequencing of ion-channel and cardiomyopathy-related genes has identified pathogenic or likely pathogenic variants in up to 25–30% of autopsy-negative cases [15]. Subsequent family cascade screening not only clarifies the cause of death but also identifies at-risk relatives, enabling preventive interventions. However, the expansion of genetic testing raises ethical concerns regarding incidental findings, consent, and data governance, which remain unevenly regulated across forensic systems [16,17,18].

These developments converge toward the broader concept of multi-omics forensic medicine, in which protein, peptide, genetic, transcriptomic, and metabolomic data are integrated into unified, mechanism-oriented diagnostic frameworks [20]. The present review provides a comprehensive synthesis of post-mortem biomarkers in SCD, spanning classical protein and peptide indicators, inflammatory and immunohistochemical markers, molecular autopsy, and multi-omics integration. Particular emphasis is placed on pre-analytical and post-analytical challenges, medico-legal implications, and future perspectives, with the aim of progressing toward standardized and internationally harmonized protocols for the forensic investigation of sudden cardiac death.

## 2. Protein Biomarkers of Myocardial Injury

Protein biomarkers remain the cornerstone of post-mortem biochemistry in sudden cardiac death (SCD), representing the first line of evidence when structural or histological signs of ischemia are absent. The most investigated molecules are cardiac troponins (cTnI and cTnT), creatine kinase-MB (CK-MB), heart-type fatty acid-binding protein (H-FABP), and glycogen phosphorylase isoenzyme BB (GPBB). Their forensic application reflects decades of clinical validation in acute coronary syndromes, yet translation to post-mortem diagnostics requires careful consideration of redistribution, hemolysis, post-mortem interval (PMI), and matrix variability. Despite these challenges, recent studies have refined our understanding of post-mortem behavior and highlighted the value of multimarker strategies that significantly improve diagnostic reliability compared with single-analyte assays [19].

Troponins are generally accepted as the most specific and sensitive markers of cardiomyocyte necrosis. In forensic settings, their measurement in pericardial fluid and femoral blood consistently demonstrates diagnostic utility. Several authors reported that pericardial troponin concentrations remain stable for longer periods than blood values, making them particularly useful when PMI exceeds 24–48 h [20]. More recent high-sensitivity assays (hs-cTnT and hs-cTnI) have broadened the post-mortem diagnostic window, enabling detection of subtle myocardial injury that might otherwise escape recognition at routine histology [21]. Importantly, some groups have proposed interpretive models based not on absolute concentrations—highly variable in post-mortem conditions—but on ratios such as pericardial fluid-to-femoral blood troponin values, which minimize the influence of PMI and hemolysis [22]. This transition from rigid cut-offs to relative or probabilistic models reflects a broader methodological evolution in forensic biochemistry.

Although troponins dominate the field, CK-MB remains a marker of interest, albeit with known limitations. Its concentration may increase in cardiac deaths but also in trauma, seizures, and muscular injury, reducing specificity [23]. Nonetheless, combined interpretation with troponins may provide additive value. For example, concordant elevation of cTnT and CK-MB in femoral blood has been associated with acute ischemic death within short PMIs, supporting a true cardiac origin rather than an artifact [24]. Recent observations suggest that CK-MB may also be informative in exertional deaths, where muscular involvement complicates interpretation; in these cases, its distinct release kinetics may reveal diagnostic patterns not captured by troponins [25].

H-FABP, a small cytosolic protein released rapidly after ischemic injury, has emerged as a promising early marker. Forensic studies demonstrate elevated H-FABP levels in blood and pericardial fluid of ischemic deaths compared with controls, particularly when survival after ischemia was extremely short [26]. However, its diagnostic reliability decreases with advancing PMI because of rapid proteolytic degradation. Recent metabolomic investigations indicate that H-FABP correlates with specific alterations in myocardial energy metabolism, suggesting that in future applications it may serve not only as a marker of necrosis but also as an indicator of metabolic collapse, potentially distinguishing ischemic from arrhythmic deaths lacking overt necrosis [27].

GPBB has received less attention but remains relevant as an indicator of early ischemic stress. Studies consistently report higher GPBB levels in ischemic SCD compared with non-cardiac deaths [28], although reproducibility varies across laboratories, likely due to methodological heterogeneity. Recent research combining GPBB with H-FABP and troponins into multimarker panels has demonstrated improved sensitivity for detecting very early ischemia when histology is negative [29]. Limitations persist, notably the lack of commercial assays optimized for post-mortem samples. Interestingly, experimental data suggest that GPBB may also reflect glycogen metabolism disturbances in sudden death, potentially linking biochemical patterns with histological features such as contraction band necrosis [28].

Overall, protein biomarkers remain essential in the forensic investigation of SCD. The most relevant recent advance is the shift toward multimarker panels and probabilistic interpretation, where combinations of necrosis, stress, and metabolic biomarkers—adjusted for PMI and sampling site—substantially outperform single-marker analyses. This trend reflects a broader transition in forensic science from reliance on isolated biochemical indicators to integrated profiles capable of withstanding both scientific scrutiny and medico-legal evaluation [30,31].

When considered collectively, protein biomarkers differ markedly in diagnostic accuracy, post-mortem stability, cost, and practical feasibility. Troponins remain the most robust and specific indicators of cardiomyocyte necrosis, although their concentration is still influenced by hemolysis and post-mortem interval. CK-MB is inexpensive and widely available but suffers from low specificity due to skeletal muscle cross-reactivity. H-FABP and GPBB disclose very early ischemic changes but degrade rapidly and require well-preserved samples. As a whole, their combined interpretation offers greater reliability than any individual marker, yet their practical use must always be weighed against matrix quality, laboratory resources, and the experience of the forensic team.

## 3. Peptidic Biomarkers and Hemodynamic Stress

While protein markers primarily reflect direct myocardial necrosis, peptide biomarkers provide insights into hemodynamic stress, neurohormonal activation, and ventricular remodeling—processes that often precede or accompany sudden cardiac death (SCD). Among these, B-type natriuretic peptide (BNP) and its inactive fragment NT-proBNP remain the most extensively studied, but forensic research has increasingly focused on copeptin, a stable surrogate of vasopressin release, and soluble ST2 (sST2), a marker of myocardial strain and fibrosis. Together, these peptides complement necrosis markers by capturing the systemic and mechanical dimensions of cardiac failure, thereby offering a broader interpretive framework for understanding the pathophysiology of SCD in post-mortem analyses [32].

BNP and NT-proBNP are released in response to ventricular wall stretch and are widely used clinically to diagnose and monitor heart failure. In forensic practice, they have been detected in blood and pericardial fluid, with the latter proving more reliable due to its protection from post-mortem redistribution. Several authors have reported significantly elevated pericardial NT-proBNP concentrations in deaths due to chronic heart failure, hypertrophic cardiomyopathy, and dilated cardiomyopathy, even when histology was inconclusive [33]. NT-proBNP also demonstrates relative post-mortem stability for at least 24–48 h, and some groups have proposed tentative interpretive ranges to differentiate cardiac from non-cardiac fatalities [34]. More recent studies have expanded beyond quantification alone: immunohistochemical detection of BNP in myocardial tissue has been correlated with ventricular hypertrophy and fibrosis, reinforcing the role of natriuretic peptides not only as circulating biomarkers but also as molecular indicators of chronic cardiac remodeling [35]. Nevertheless, interpretation remains challenging in the presence of comorbidities such as renal failure or pulmonary hypertension, which may independently elevate natriuretic peptide levels and thereby necessitate multiparametric approaches.

Copeptin has recently emerged as a particularly attractive biomarker for forensic applications. Derived from the C-terminal portion of the vasopressin precursor, it reflects acute stress and hemodynamic collapse. A major advantage of copeptin is its biochemical stability: unlike vasopressin, it can be reliably measured in femoral blood and vitreous humor across a range of post-mortem intervals. Several studies demonstrated that copeptin levels are significantly higher in ischemic sudden deaths than in traumatic or non-cardiac controls, supporting its potential as a discriminator of acute cardiac events. More recently, multimarker investigations showed that combining copeptin with high-sensitivity troponins and early necrosis markers such as H-FABP can achieve diagnostic accuracies exceeding 90% for ischemic SCD [7,36]. This synergy highlights the conceptual value of copeptin in forensic practice: it does not replace troponins but captures the systemic neurohormonal response, bridging the mechanistic gap between ischemia and stress-related cardiac collapse. However, copeptin is not specific to cardiac pathology and may rise in sepsis, trauma, and stroke, making contextual interpretation essential.

sST2, a member of the interleukin-1 receptor family, reflects myocardial strain, fibrosis, and adverse remodeling. Although well validated clinically as a prognostic marker in heart failure, forensic studies are still limited but increasingly promising. Some investigations reported significantly higher sST2 concentrations in pericardial fluid in ischemic SCD compared with non-cardiac deaths, supporting its role as an indicator of acute myocardial strain [32]. Emerging data also suggest that pairing sST2 with NT-proBNP enhances diagnostic reliability, particularly in cases involving chronic structural heart disease where distinguishing terminal arrhythmia from progressive decompensation is challenging [37]. Additional peptides such as mid-regional pro-adrenomedullin (MR-proADM) and mid-regional pro-atrial natriuretic peptide (MR-proANP) have been measured in post-mortem fluids, and preliminary reports link them to circulatory failure and systemic stress, though validation in large forensic cohorts remains pending [38].

The most significant development in recent years lies not in any single peptide but in the adoption of multimarker panel strategies. Several studies have shown that combinations of NT-proBNP, copeptin, high-sensitivity troponins, H-FABP, and inflammatory mediators such as pentraxin-3 achieve far higher diagnostic performance than individual markers [39]. These composite models integrate necrosis, stress, and inflammatory biomarkers into a unified interpretive approach, reflecting the multifactorial mechanisms underlying sudden death. Their integration with computational tools—including machine-learning algorithms recently applied in forensic metabolomics—represents a promising frontier in which biochemical evidence is interpreted probabilistically rather than through rigid concentration thresholds [40].

When considered collectively, peptide biomarkers differ markedly in diagnostic accuracy, post-mortem stability, and specificity. NT-proBNP is highly informative in chronic remodeling but less specific in renal or pulmonary disease; copeptin offers excellent stability and insight into acute stress responses but lacks disease specificity; and sST2 reflects structural myocardial strain but requires specialized assays and remains less validated in forensic settings. Their greatest diagnostic strength lies in combined, multimarker interpretation, which consistently outperforms isolated peptide assessment and aligns with the broader transition toward integrated, mechanistic approaches in forensic cardiopathology.

## 4. Inflammatory and Immunohistochemical Biomarkers

Inflammation is increasingly recognized as a pivotal component of sudden cardiac death (SCD), acting both as a primary mechanism—as in myocarditis, autoimmune disease, or systemic inflammatory syndromes—and as a secondary driver of myocardial ischemia and arrhythmogenic vulnerability. From a forensic perspective, the assessment of inflammatory pathways through soluble biomarkers and immunohistochemistry (IHC) provides valuable complementary information when autopsy and routine histology are inconclusive. While soluble mediators such as cytokines may support diagnostic hypotheses, their non-specificity and susceptibility to post-mortem changes mandate cautious interpretation. In contrast, tissue-based IHC markers offer greater specificity by demonstrating vital reactions and allowing a more precise temporal staging of myocardial injury [41].

Among soluble inflammatory markers, C-reactive protein (CRP) and interleukin-6 (IL-6) are the most studied. Several authors reported significantly higher CRP levels in femoral blood in deaths related to myocarditis and sepsis than in ischemic sudden deaths, suggesting that CRP may assist in distinguishing inflammatory from ischemic mechanisms [42]. Likewise, IL-6 concentrations have been shown to rise in acute inflammatory deaths, correlating with histological evidence of leukocytic infiltration [43]. However, both CRP and IL-6 can be elevated in systemic hypoxia, prolonged agony, or terminal stress, reducing their forensic specificity. Recent multiparametric approaches combining cytokines with necrosis markers—such as IL-6, troponins, and NT-proBNP—may enhance diagnostic reliability in myocarditis-related deaths [10].

The most substantial advances in this field have come from IHC. Fibronectin deposition along the sarcolemma is now recognized as one of the earliest markers of ischemic injury. Several studies demonstrated that fibronectin immunostaining can reveal ischemic damage within one hour of coronary occlusion, at a stage when hematoxylin–eosin staining remains negative [44]. Complement activation, particularly deposition of C5b-9 (the membrane attack complex), also provides strong discriminatory power, demonstrating cardiomyocyte vitality at the time of ischemia; complement deposition has been consistently detected in acute ischemic deaths but is absent in non-ischemic or chronic conditions [45]. Additional markers such as desmin disruption and S100A1 depletion further assist in identifying early ischemia. Loss of S100A1 immunoreactivity can occur within one hour of ischemia, offering one of the earliest molecular indicators of myocardial injury [46].

Systemic inflammatory and autoimmune diseases have also gained attention as contributors to sudden death. Systemic sclerosis has been associated with diffuse myocardial fibrosis and microvascular dysfunction culminating in fatal arrhythmias, while cardiac sarcoidosis remains an important cause of unexplained sudden death, frequently revealing granulomatous infiltration of the conduction system in cases initially classified as autopsy-negative [47,48]. Infectious etiologies remain relevant: during the COVID-19 pandemic, endothelial infection of coronary microvasculature by SARS-CoV-2 was documented in fatal cases, linking viral invasion with sudden collapse even in the absence of overt myocarditis [49]. In pediatric populations, Kawasaki disease and multisystem inflammatory syndrome in children (MIS-C) have been associated with fatal arrhythmias and coronary vasculitis, underscoring the diverse inflammatory pathways leading to SCD [50].

New molecular pathways and imaging correlates are also being investigated. Li et al. demonstrated upregulation of nuclear factor-κB (NF-κB) and activating transcription factor 3 (ATF3) in myocardial samples from autopsy-negative SCD, suggesting that subtle inflammatory processes may remain histologically silent [51]. The pericoronary fat attenuation index (FAI), an imaging biomarker of coronary inflammation, has been shown in post-mortem CT angiography that inflamed coronary segments can be identified non-invasively, providing a bridge between forensic imaging and molecular pathology [52].

Taken together, inflammatory and IHC biomarkers represent an essential extension of the forensic diagnostic toolkit for SCD. Soluble mediators such as CRP and IL-6 offer supportive, though non-specific, evidence of inflammation, whereas fibronectin deposition, complement activation, desmin disruption, and S100A1 depletion provide robust IHC markers of acute ischemic injury. Systemic autoimmune and infectious conditions highlight the need for broad diagnostic perspectives, as SCD may arise from complex multisystem processes. Emerging molecular markers and imaging-based approaches point toward a future in which biochemistry, histology, and radiology converge, producing more reliable reconstructions of inflammatory contributions to sudden cardiac death.

When compared side-by-side, inflammatory biomarkers display highly variable forensic performance. Soluble cytokines are accessible and inexpensive but prone to post-mortem degradation and bacterial translocation, limiting their specificity. Conversely, IHC markers offer superior evidence of vital reactions and ischemic timing but require specialized technical expertise and longer processing times. A critical comparison shows that IHC integrated with histology provides the strongest diagnostic value, whereas soluble inflammatory markers function best as complementary elements within a broader, multimodal interpretive framework.

## 5. MicroRNAs and Molecular Biomarkers

The last decade has witnessed an extraordinary expansion in the study of molecular biomarkers for sudden cardiac death (SCD), with microRNAs (miRNAs), exosomal nucleic acids, and proteomic and metabolomic profiles emerging as innovative tools that extend far beyond classical protein and peptide assays. Unlike traditional markers, these molecules provide insight into gene regulation, cell signaling, and systemic metabolic collapse, thereby addressing the crucial problem of autopsy-negative sudden unexplained death. Their value lies not only in diagnostic discrimination but also in revealing mechanisms of death that remain invisible to histology and routine biochemistry [53].

MicroRNAs are short non-coding RNAs that regulate gene expression post-transcriptionally and are released into circulation during tissue injury. They are particularly attractive for forensic applications because of their stability in post-mortem samples and their tissue specificity. Several studies demonstrated that cardiac miR-1, miR-133, miR-208, and miR-499 are significantly elevated in ischemic sudden deaths, correlating with troponin release and histological evidence of necrosis [54]. More recent investigations highlighted that distinct miRNA signatures can distinguish ischemic deaths from arrhythmic sudden deaths without structural changes, making them powerful adjuncts in autopsy-negative cases [55]. Importantly, high-throughput sequencing and quantitative PCR have confirmed that miRNA profiles remain stable across post-mortem intervals of up to 72 h, further reinforcing their applicability in forensic settings [56].

A particularly promising development has been the study of exosomal miRNAs and extracellular vesicles, which protect nucleic acids from degradation. Exosomal miRNAs have shown superior discriminatory ability in differentiating ischemic sudden deaths from non-cardiac fatalities compared with plasma miRNAs alone [57]. Huang et al. further demonstrated that exosomal signatures could distinguish ischemic cardiomyopathy, hypertrophic cardiomyopathy, and arrhythmic deaths, suggesting that extracellular vesicles may encode phenotype-specific molecular profiles [58]. The encapsulation of nucleic acids within vesicles also enhances long-term stability, opening opportunities for retrospective analyses of stored forensic material.

Beyond miRNAs, proteomic and metabolomic investigations have opened new diagnostic horizons. Proteomic studies identified differential expression of mitochondrial enzymes, annexins, and heat shock proteins in ischemic versus non-ischemic deaths, pointing to stress-induced alterations at the cellular machinery level. Metabolomics has revealed consistent shifts in acylcarnitines, amino acids, and tricarboxylic acid cycle intermediates, reflecting profound mitochondrial dysfunction during fatal cardiac arrest. Importantly, these metabolic fingerprints evolve in predictable ways with the post-mortem interval, indicating that PMI-adjusted interpretive models are essential for reliable forensic use. This represents a significant conceptual advancement: biomarkers are no longer interpreted as static values but as components of dynamic temporal models.

The integration of multilayer molecular data has led to the emergence of multi-omics approaches that combine genomics, transcriptomics, proteomics, and metabolomics into unified diagnostic frameworks. Recent studies applying machine learning to these datasets have achieved remarkable results, with several reports documenting over 90% sensitivity and specificity in distinguishing ischemic from non-ischemic sudden deaths [59,60,61,62]. This shift toward AI-assisted forensic cardiology marks a substantial methodological evolution: rather than relying on isolated markers, post-mortem investigations increasingly adopt probabilistic, data-driven classification models capable of interpreting complex molecular patterns. Although still experimental, these approaches suggest that the future of forensic pathology will increasingly mirror precision medicine, integrating diverse data streams to support individualized case interpretation.

In conclusion, molecular biomarkers—particularly miRNAs and exosomal RNA profiles—represent one of the most dynamic and transformative frontiers in forensic medicine. Classical cardiac miRNAs provide reliable evidence of myocardial injury; exosomal miRNAs enhance biomarker stability and disease specificity; and proteomic and metabolomic signatures offer broader insight into the systemic collapse underlying sudden death. The incorporation of multi-omics strategies and AI-driven classification further elevates diagnostic potential, but implementation in routine practice requires multicenter validation, standardization of analytical workflows, and consensus guidelines. As the field evolves, molecular biomarkers are poised not only to clarify autopsy-negative deaths but also to redefine the investigative paradigm of sudden cardiac death [53,54,55,56,57,58,59,60,61,62].

More recent studies have expanded the spectrum of post-mortem biomarkers relevant to sudden cardiac death. Evidence has emerged that the post-mortem stability of troponin and CK-MB may be less satisfactory than previously assumed, especially in advanced decomposition stages or prolonged intervals [63]. Proteomic investigations have identified additional candidates such as MYH6 and COX5B, which appear to reflect mitochondrial and contractile dysfunction in acute ischemic collapse [64]. Similarly, variants in CACNA1A, traditionally associated with neurological disorders, have recently been implicated in arrhythmogenic vulnerability and fatal cardiac events [65]. Endoplasmic-reticulum stress–related secretory proteins have also been recognized as potential indicators of cardiomyocyte distress and may offer new opportunities for early detection of ischemic or metabolic failure [66]. Incorporating these emerging biomarkers highlights the dynamic evolution of the field and underscores the importance of continuous updates as new evidence becomes available.

Across protein, peptide, inflammatory, and molecular biomarkers, reported sensitivity and specificity values vary substantially between studies, largely reflecting heterogeneity in case selection, analytical techniques, and definitions of reference standards. Several promising biomarkers have been assessed in relatively small cohorts or under controlled experimental conditions, whereas their diagnostic performance in real-world forensic settings is often less robust. Reproducibility across laboratories remains a significant challenge, especially for emerging methods such as miRNA profiling, exosomal RNA analysis, and untargeted proteomic or metabolomic platforms. These observations underscore the need for multicenter validation, harmonization of analytical protocols, and external quality assessment before any biomarker panel can be considered suitable for standardized forensic implementation.

## 6. Molecular Autopsy and Genetic Insights

While Section 5 focuses on molecular biomarkers that capture functional and regulatory disturbances at the RNA, protein, and metabolic levels, the present section addresses genetic variants identified through DNA-based approaches. This distinction reflects two complementary but conceptually separate layers of biological investigation. Molecular biomarkers describe dynamic cellular responses occurring in the peri-mortem phase, whereas molecular autopsy targets stable genomic alterations that predispose to lethal arrhythmogenic or cardiomyopathic phenotypes. Although both domains contribute to the broader multi-omics landscape, maintaining this separation improves clarity by distinguishing mechanisms of injury from inherited genetic susceptibility.

One of the most transformative innovations in the investigation of sudden cardiac death (SCD) has been the introduction of the molecular autopsy, which refers to the post-mortem use of genetic testing to identify pathogenic variants associated with inherited cardiac disorders. Traditional autopsy and histology, while indispensable, cannot detect electrical or molecular abnormalities that often underlie unexplained sudden deaths, particularly in young individuals with structurally normal hearts. Over the last decade, advances in next-generation sequencing (NGS) technologies have expanded the scope of genetic investigations from targeted panels to whole-exome sequencing (WES) and even whole-genome sequencing (WGS), allowing systematic exploration of cardiac channelopathies and cardiomyopathies as potential causes of unexplained fatalities [67].

Channelopathies represent a major target of molecular autopsy, as they frequently present without morphological abnormalities. Long QT syndrome (LQTS), Brugada syndrome (BrS), catecholaminergic polymorphic ventricular tachycardia (CPVT), and short QT syndrome (SQTS) are among the most common arrhythmic syndromes associated with sudden death. The most frequently implicated genes include *SCN5A*, *KCNQ1*, *KCNH2*, and *RYR2*, which encode sodium, potassium, and calcium channel subunits essential for cardiac electrophysiology [68]. Several studies reported pathogenic or likely pathogenic variants in these genes in approximately 20–30% of autopsy-negative sudden unexplained deaths, confirming their significance in forensic settings. However, variants of uncertain significance (VUS) represent a persistent challenge, accounting for up to half of detected changes. Interpretation requires functional assays, computational modeling, and population frequency analysis, yet inter-laboratory variability complicates medico-legal conclusions [69,70].

Cardiomyopathies constitute another crucial category in which molecular autopsy has yielded important insights. Genetic variants in desmosomal proteins (e.g., PKP2, DSP, DSG2, DSC2) are strongly associated with arrhythmogenic right ventricular cardiomyopathy (ARVC), while mutations in sarcomeric and cytoskeletal genes (e.g., *MYH7*, *LMNA*, *DES*) are linked to hypertrophic and dilated cardiomyopathies. In multiple forensic series, pathogenic variants in cardiomyopathy-related genes were identified in victims whose hearts appeared grossly normal at autopsy, suggesting that genetic predisposition may precede structural manifestations and trigger fatal arrhythmias [71]. A recent multicenter study demonstrated that combining cardiomyopathy-related genes with channelopathy panels increased the overall diagnostic yield of molecular autopsy by nearly 40%, supporting broad sequencing strategies rather than narrow gene panels [72].

Beyond determining the cause of death, molecular autopsy has significant implications for surviving relatives. Identifying a pathogenic variant enables cascade screening, allowing family members to undergo genetic counseling, clinical surveillance, and preventive interventions [73]. Systematic family screening after autopsy-negative sudden death has revealed previously unrecognized carriers, preventing additional fatalities [74]. However, this also raises ethical considerations involving consent, incidental findings, privacy of genetic data, and the duty to inform relatives—issues governed differently across jurisdictions.

A novel evolution in molecular autopsy is the incorporation of multi-omics approaches. While NGS identifies DNA-level alterations, transcriptomics, proteomics, and metabolomics uncover functional consequences. Recent studies integrating transcriptomic signatures with genetic data have improved classification of unexplained SCD, particularly in cases lacking identifiable pathogenic variants [75,76]. Machine-learning algorithms leveraging multi-omics datasets have achieved diagnostic accuracies exceeding 90%, heralding the emergence of AI-driven genomic forensics [77]. Although experimental, these models reflect a shift toward multidimensional, data-driven interpretation.

A persistent challenge is the high prevalence of VUS, which cannot be easily categorized as pathogenic or benign. Their interpretation requires integration of population genetics, in silico predictive tools, functional assays, segregation analysis, and phenotypic context. The complexity of VUS assessment underscores the importance of molecular autopsy boards, multidisciplinary committees involving forensic pathologists, cardiologists, clinical geneticists, molecular biologists, and ethicists, which enhance both scientific rigor and medico-legal defensibility.

To facilitate interpretation, Table 1 summarizes principal gene categories associated with sudden unexplained death, including well-established channelopathy and cardiomyopathy genes as well as emerging susceptibility loci such as *MYH6*, *COX5B,* and *CACNA1A* (Table 1 and Table 2).

## 7. Pre-Analytical and Post-Analytical Considerations

The interpretation of post-mortem biomarkers in sudden cardiac death (SCD) is strongly influenced by pre-analytical and post-analytical variables, which often determine the difference between reliable evidence and misleading results. Unlike clinical biochemistry, where blood is sampled under standardized conditions, post-mortem samples are exposed to autolysis, putrefaction, hemolysis, and microbial proliferation. Consequently, the diagnostic power of biomarkers depends not only on their intrinsic specificity or sensitivity but also on how rigorously samples are collected, preserved, analyzed, and interpreted [78].

Among pre-analytical factors, the choice of biological matrix plays a decisive role. Femoral blood is generally preferred because it is less affected by redistribution, whereas central cardiac blood may show artificially elevated values due to local myocardial leakage [79]. Pericardial fluid is increasingly recognized as a reliable alternative, particularly for NT-proBNP and troponins, owing to its anatomical isolation and stability [32]. Vitreous humor, traditionally used for metabolites, has recently proven useful for copeptin and other peptides because it resists hemolysis and putrefaction [80]. Cerebrospinal fluid has also been explored for cytokines and miRNAs, although its use remains less standardized [81].

Post-mortem interval (PMI) is another fundamental variable. Biomarker concentrations change predictably with increasing PMI: cardiac troponins may remain measurable for over 48 h, while small cytosolic proteins such as H-FABP degrade more rapidly [82]. Recent metabolomic studies demonstrated systematic PMI-related shifts in acylcarnitines, amino acids, and tricarboxylic acid intermediates, supporting the development of PMI-adjusted interpretive models [83]. Similarly, although miRNAs and exosomal RNAs generally display excellent stability, sequence-dependent differences underline the need for specific validation of each candidate biomarker [84].

Sample collection and storage protocols remain essential for reproducibility. Current recommendations from the AECVP and SCVP emphasize aseptic femoral sampling, preferably with fluoride-containing tubes to limit glycolysis, followed by centrifugation and storage at −80 °C for molecular assays [85]. Early aspiration of pericardial fluid minimizes contamination, and dried blood spot (DBS) cards have recently been validated as a cost-effective tool for long-term DNA and RNA preservation [86]. Hemolysis can artificially increase CK-MB and LDH, while bacterial translocation may elevate cytokines such as IL-6 and CRP, potentially mimicking inflammatory disease [87,88]. Visual inspection and exclusion of compromised samples remain indispensable.

Post-analytical interpretation is equally crucial. Universal cut-offs used in clinical medicine cannot be directly applied in the post-mortem setting because concentrations vary widely by matrix, PMI, and comorbidities. Relative measures, such as pericardial fluid-to-blood ratios for troponins or NT-proBNP, often provide more robust information than absolute concentrations [89]. Recent approaches—such as Bayesian modeling and machine-learning algorithms—combine biomarker data with metadata (age, PMI, comorbidities) to generate probabilistic rather than deterministic conclusions, a strategy that better reflects the inherent uncertainty of forensic interpretation and aligns with medico-legal standards requiring transparent evaluation of reliability [90].

Taken together, matrix choice, PMI-adjusted interpretation, rigorous sampling and storage protocols, and awareness of contamination sources represent overarching modifiers that influence all post-mortem biochemical and molecular analyses. Rather than reiterating these factors for every individual biomarker, they should be considered a unifying framework within which all assays must be contextualized. At the same time, emerging probabilistic and multimarker models—supported by artificial intelligence—are gradually replacing rigid interpretive thresholds. The standardization of protocols and multicenter validation remain urgent priorities to ensure that biomarker evidence withstands scientific scrutiny and judicial evaluation [32,78,79,80,81,82,83,84,85,86,87,88,89,90] (Table 3).

## 8. Forensic and Legal Implications

The use of post-mortem biomarkers in sudden cardiac death (SCD) enriches not only scientific investigation but also carries significant forensic and legal implications. In malpractice litigation, insurance disputes, and criminal trials, the ability of biomarkers to clarify the cause of death can decisively influence judicial outcomes. However, their admissibility and reliability depend on methodological rigor, standardized interpretation, and explicit acknowledgment of their inherent limitations by forensic experts [39].

A major issue is the absence of universally accepted post-mortem cut-offs. In clinical practice, validated thresholds exist for troponins, BNP, and other biomarkers, but these values cannot be directly applied post-mortem due to redistribution, hemolysis, and variability in post-mortem intervals. As a result, courts may hesitate to admit biomarker evidence in the absence of clear interpretive standards. Recent studies have shown that PMI-adjusted models combining metabolomic signatures with protein biomarkers significantly improve diagnostic accuracy, suggesting that probabilistic rather than absolute interpretive approaches may be more defensible in judicial settings [91]. This shift aligns with the Daubert criteria for admissibility of scientific evidence, which emphasize reproducibility, peer review, and transparent error rates.

Hospital-related deaths represent another important medico-legal context. When patients collapse shortly after admission and autopsy reveals no clear lesions, biomarker analysis may help support or refute allegations of delayed recognition of myocardial infarction or inadequate treatment. For example, Kutlu et al. reported that elevated copeptin alongside troponins in post-mortem blood indicated acute ischemia at the time of collapse, enabling retrospective reconstruction of a missed clinical diagnosis [92]. Conversely, normal biomarker levels in similar contexts may protect clinicians from unjustified accusations by demonstrating that the death resulted from a sudden arrhythmic event beyond medical control. In this way, biomarkers act as double-edged tools in malpractice litigation, with the potential to support both prosecution and defense.

Biomarkers also play a growing role in insurance and civil law. Several studies have shown that including biomarker data in forensic autopsy reports reduces the proportion of unexplained deaths in disputed insurance cases, thereby facilitating resolution and accelerating compensation for families [15]. The ethical and legal implications of molecular autopsy deserve particular consideration. Because pathogenic variants detected in the deceased may indicate hereditary risk in surviving relatives, post-mortem genetic testing has a societal dimension that extends beyond the immediate case. Some studies reported that family screening initiated after molecular autopsy prevented additional sudden deaths in carriers of arrhythmic syndromes, highlighting the public health value of forensic genetics [93]. Yet, this benefit raises questions regarding consent, privacy, data ownership, and disclosure: who controls genetic information after death, and who bears responsibility for informing at-risk relatives? Legal frameworks differ substantially across jurisdictions, with some mandating disclosure and others leaving decisions to forensic authorities, resulting in heterogeneous practices that can affect public health and legal proceedings [94].

Finally, the introduction of artificial intelligence (AI) into forensic workflows is poised to reshape medico-legal interpretation. Recent studies reported that machine-learning models trained on multi-omics biomarkers achieved diagnostic accuracies exceeding 90% in distinguishing ischemic SCD from controls, suggesting that AI-derived probabilities may eventually be presented as expert evidence [95]. However, legal systems will require transparency, explainability, and independent validation before accepting AI-generated outputs in court. This tension between technological innovation and judicial conservatism is likely to shape forensic discourse for years to come.

**Figure 1:**
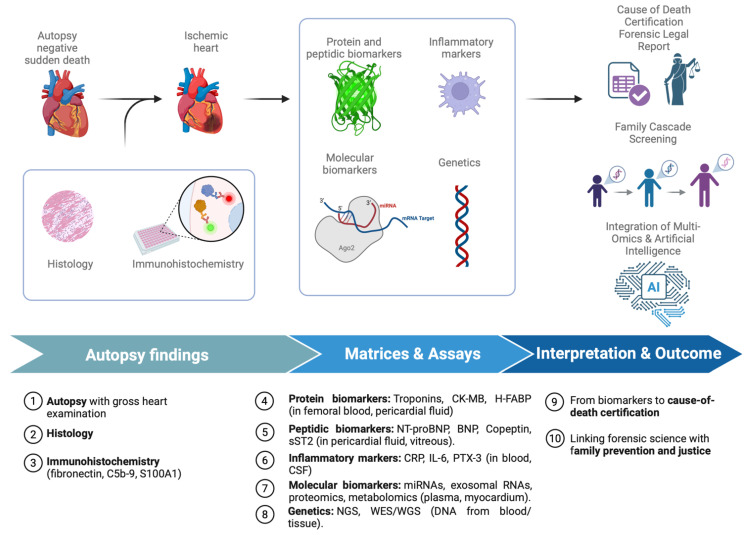
Integrated Biomarker Workflow in Sudden Cardiac Death Investigation. Created in BioRender. Aquila, I. (2026) https://BioRender.com/lqdfht8.

In conclusion, the forensic and legal implications of biomarker research in SCD are broad and multifaceted. Biomarkers can strengthen medico-legal diagnoses, clarify liability in malpractice litigation, resolve insurance disputes, and guide preventive interventions in families. At the same time, their probative value depends on standardization, validation, and ethical governance. The challenge ahead is to translate scientific advances into protocols capable of withstanding judicial scrutiny, ensuring that biomarkers function as reliable bridges between forensic science and the law [15,39,91,92,93,94]. These medico-legal challenges arise within broader international frameworks governing genetic data protection. Instruments such as the Council of Europe’s Oviedo Convention, the European Union’s General Data Protection Regulation (GDPR), and national regulations on genetic nondiscrimination (such as the U.S. Genetic Information Nondiscrimination Act) illustrate the diversity of approaches to genetic privacy and data governance. Although not specifically designed for forensic molecular autopsy, these frameworks provide essential reference principles regarding consent, proportionality, and secondary use of genetic information, and should inform the development of dedicated guidelines for post-mortem practice (Figure 1).

## 9. Future Perspectives and Research Agenda

The field of post-mortem biomarkers in sudden cardiac death (SCD) is rapidly evolving, and the coming years are likely to witness profound changes in both the scientific and forensic landscapes. While current practice still relies heavily on isolated biomarkers such as troponins or NT-proBNP, the recent literature consistently highlights that no single analyte can provide conclusive evidence in isolation. Instead, future diagnostic strategies will increasingly depend on multimarker panels integrating indicators of necrosis, hemodynamic stress, inflammation, and molecular alterations into a unified interpretive framework [95]. This comprehensive approach better reflects the multifactorial pathophysiology of SCD and offers enhanced robustness in medico-legal contexts, where conclusions must withstand cross-examination and judicial scrutiny.

The emergence of multi-omics strategies is among the most promising developments. Proteomic and metabolomic analyses have begun to reveal reproducible molecular fingerprints of ischemic cardiac death, while transcriptomic and miRNA profiling contribute additional layers of specificity [60]. When combined, these high-dimensional datasets provide a multidimensional reconstruction of myocardial injury, arrhythmic susceptibility, and systemic collapse. Integrating these diverse data streams into interpretable forensic tools represents a major challenge, yet early applications of artificial intelligence have shown the potential to achieve diagnostic accuracies exceeding 90% in experimental settings [77]. Nevertheless, incorporating AI into routine forensic workflows will require rigorous technical validation, ethical oversight, and clear legal frameworks capable of ensuring transparency and accountability.

Standardization and harmonization of forensic biomarker protocols constitute another essential area for future progress. Significant heterogeneity persists across laboratories regarding sampling sites, storage conditions, analytical methods, and interpretive thresholds. This variability undermines reproducibility and weakens the admissibility of biomarker evidence in court. Although organizations such as the Association for European Cardiovascular Pathology (AECVP) and the Society for Cardiovascular Pathology (SCVP) have issued general autopsy guidelines, biomarker-specific protocols remain fragmented and incomplete [96,97]. The development of international consensus guidelines—particularly concerning post-mortem use of troponins, natriuretic peptides, cytokines, and molecular assays—should therefore be prioritized. Multicenter collaborative studies establishing reference ranges adjusted for post-mortem interval and biological matrix will be crucial to achieving clinically and legally credible standards.

The expansion of molecular autopsy represents an additional frontier. As sequencing costs decline and analytic pipelines improve, incorporating whole-exome and whole-genome sequencing into routine forensic workflows is becoming increasingly feasible. Functional assays to clarify variants of uncertain significance will likely enhance diagnostic yield in autopsy-negative deaths [98]. At the same time, the public health implications of molecular autopsy are becoming more widely recognized: identifying pathogenic variants in the deceased enables family cascade screening and targeted preventive strategies, transforming forensic findings into tools with societal impact [99]. Realizing this potential requires clear ethical and legal frameworks governing consent, data sharing, and communication of genetic results to relatives.

Emerging technologies are poised to further expand the forensic toolkit. Imaging biomarkers such as the pericoronary fat attenuation index (FAI), derived from post-mortem CT angiography, have been proposed as non-invasive indicators of coronary inflammation [52]. Convergence between digital pathology, virtual autopsy, AI-driven image analysis, and molecular assays may eventually yield comprehensive multimodal workflows. The long-term vision is the development of a “digital-molecular autopsy,” in which imaging, histology, biochemistry, genomics, and computational modeling are integrated into a cohesive diagnostic narrative of sudden death.

Looking ahead, the research agenda must prioritize translational studies bridging the gap between clinical and forensic cardiology. Many biomarkers validated in living patients have not yet been systematically evaluated post-mortem, and forensic discoveries often lack clinical validation. Collaborative networks involving forensic institutes, cardiology departments, and molecular research laboratories will be essential to generate large, diverse datasets suitable for meta-analyses and robust evidence generation [100]. Because sudden cardiac death carries profound medico-legal and public health implications, sustained investment from funding agencies and policymakers is crucial.

Recent advances in artificial intelligence and machine learning have further revealed both promising opportunities and significant challenges for post-mortem cardiac diagnostics. The development of reliable predictive tools requires large, diverse, and rigorously annotated datasets capable of representing the heterogeneity of forensic populations. Insufficient representation risks introducing algorithmic bias and may compromise medico-legal reliability. Equally critical is the “black box” nature of many deep-learning systems, which limits interpretability and may hinder admissibility in judicial settings. Future work must therefore prioritize the development of transparent, explainable models supported by standardized reporting frameworks that clarify the strengths and limitations of algorithm-derived outputs [77].

Multi-omics approaches similarly present substantial analytical and computational obstacles. Integrating genomic, transcriptomic, proteomic, and metabolomic datasets requires sophisticated bioinformatic infrastructures capable of harmonizing diverse data structures, mitigating batch effects, and accounting for post-mortem biochemical degradation. Establishing validated interpretive pipelines remains one of the most urgent unmet needs in the field.

Standardization also remains a central challenge. Forensic laboratories worldwide employ divergent sampling protocols, analytical platforms, and interpretive thresholds, reducing inter-study comparability and hindering the establishment of universally accepted reference standards. A coordinated international effort—analogous to consensus frameworks implemented in clinical oncology—will be necessary to produce detailed guidelines tailored specifically to post-mortem biomarker analysis.

The ethical and legal dimensions of molecular autopsy further demand innovative governance models. “Consent for governance” frameworks, in which families authorize controlled use and future reanalysis of post-mortem genetic data under transparent oversight mechanisms, may offer a balanced approach that reconciles privacy protection with the broader public health benefits of genetic screening. As genomic technologies continue to expand, aligning forensic workflows with such governance structures will become increasingly important.

In summary, the future of post-mortem biomarker research in SCD will depend on the convergence of multicenter validation studies, the development of transparent and explainable AI models for integrating heterogeneous datasets, and the creation of internationally coordinated guidelines that harmonize sampling, analytical methods, and interpretive criteria. Addressing these scientific, ethical, and legal challenges will be essential for transforming promising but fragmented evidence into standardized, reproducible, and judicially robust tools for the investigation of sudden cardiac death (Table 4).

## 10. Proposed Practical Framework for Post-Mortem Cardiac Diagnostics

Although significant progress has been achieved in the development of biochemical and molecular tools for the investigation of sudden cardiac death, routine forensic practice still lacks a unified diagnostic framework comparable to the structured approach used in clinical cardiology. For this reason, we propose an integrated diagnostic model that mirrors the logic of clinical workflows while adapting to the constraints and opportunities of the post-mortem setting.

The diagnostic process should begin with a careful examination of circumstantial information, which in the forensic context assumes the role played by anamnesis and symptom evaluation in clinical cardiology. Details regarding the circumstances of death, possible prodromal manifestations, physical exertion, pre-existing cardiac conditions, medication use, and family history of arrhythmic or cardiomyopathic disorders provide essential contextual elements [101]. Whenever available, information concerning resuscitation attempts or recent medical evaluations should be incorporated, as these may directly influence biochemical and morphological findings.

The subsequent autopsy stage functions as the post-mortem equivalent of first-line cardiac imaging. Gross examination of the heart allows for the identification of coronary artery disease, myocardial scarring, cardiomyopathies, and other structural abnormalities that may serve as primary substrates for sudden death. When accessible, post-mortem CT further refines structural assessment by detecting cardiomegaly, coronary calcifications, or pericardial pathology, analogous to clinical CT angiography or echocardiography [102].

Histology and immunohistochemistry represent the next fundamental diagnostic layer, corresponding to tissue-level investigations in clinical cardiology. Routine histological sections remain indispensable for recognizing myocardial necrosis, myocarditis, fibrotic remodeling, and infiltrative processes. Immunohistochemical markers such as fibronectin, C5b-9, desmin, and S100A1 provide sensitive means of detecting early ischemic changes that remain invisible to conventional staining techniques [8,103]. Viral or inflammatory panels may further assist in characterizing myocarditis or systemic inflammatory conditions, paralleling in vivo use of tissue and inflammatory biomarkers.

The integration of post-mortem biochemical analysis adds the functional dimension typically offered by circulating biomarkers in clinical medicine. The most robust and widely validated analytes remain cardiac troponins and NT-proBNP, measurable in femoral blood and pericardial fluid even with moderate post-mortem intervals [104]. Copeptin adds complementary information by reflecting acute systemic and hemodynamic stress, while soluble ST2 provides insight into myocardial strain and remodeling [59]. Additional markers such as CK-MB, H-FABP, and GPBB may be included, recognizing that combined interpretation of necrosis markers, hemodynamic stress indicators, and systemic stress mediators offers superior diagnostic reliability compared with isolated parameters [19,32,39].

In cases in which autopsy, histology, and biochemistry fail to reveal a clear cause of death, molecular analyses become indispensable, mirroring advanced testing in clinical cardiology. MicroRNA profiling—particularly of cardiac-enriched sequences such as miR-1, miR-133, miR-208, and miR-499—can distinguish ischemic mechanisms from primary arrhythmic deaths, and exosomal miRNAs may further improve diagnostic accuracy due to their increased stability [54,55,56,57,58]. When structural and biochemical explanations remain insufficient, next-generation sequencing of channelopathy- and cardiomyopathy-associated genes should be performed, representing the post-mortem parallel of clinical genetic screening [67,68,69,70,71,72,73,74,75,76,77]. In highly specialized centers, proteomic and metabolomic profiling may further elucidate metabolic dysfunction or arrhythmic susceptibility [13,27,32].

The final interpretive step requires merging all available data into a coherent, multidisciplinary reconstruction of events, similar to the integrative evaluation performed by clinical heart teams. Morphological, biochemical, molecular, and contextual findings must be weighed collectively to determine whether death resulted from ischemic injury, primary arrhythmia, cardiomyopathy, myocarditis, systemic inflammatory disease, or remains unexplained [105]. The growing availability of multimarker models and probabilistic interpretive frameworks may eventually facilitate more objective and standardized conclusions, particularly when supported by artificial intelligence tools developed for multi-omics integration [77].

**Figure 2:**
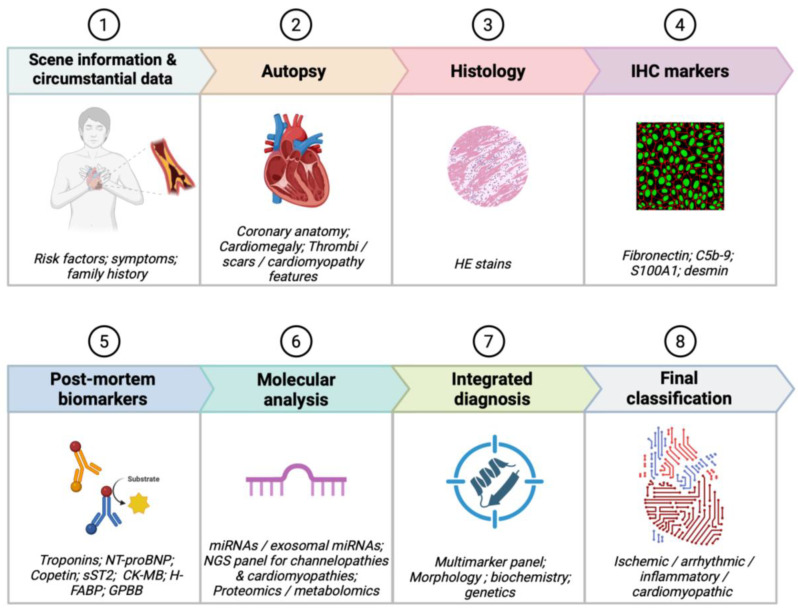
Integrated workflow for post-mortem cardiac diagnostics in sudden cardiac death (SCD). Created in BioRender. Aquila, I. (2026) https://BioRender.com/0dhmpfl.

Overall, this proposed framework provides a feasible, realistic, and clinically inspired roadmap for post-mortem cardiac diagnostics. It integrates available methods according to their accessibility, diagnostic yield, and robustness against post-mortem degradation, with the overarching goal of promoting harmonized, reproducible, and scientifically defensible practices across forensic institutions [106] (Figure 2).

## 11. Conclusions

Sudden cardiac death (SCD) remains one of the most complex and socially impactful conditions in forensic medicine, accounting for a substantial proportion of natural fatalities and often occurring in individuals without prior symptoms. Despite the central role of autopsy and histology, up to 15% of cases remain unexplained, underscoring the limitations of traditional approaches and the urgent need for complementary methods [101]. Over the last two decades, post-mortem biomarker research has provided important advances, spanning from classical protein assays to cutting-edge molecular diagnostics.

Protein biomarkers, particularly cardiac troponins, remain the most specific indicators of myocardial necrosis and continue to represent the foundation of forensic biochemistry. Their diagnostic value is enhanced when combined with additional proteins such as CK-MB, H-FABP, and GPBB, especially in multimarker strategies [8,102]. Peptide biomarkers, including NT-proBNP, copeptin, and sST2, extend this perspective by capturing hemodynamic stress and systemic neurohormonal activation, thereby refining the reconstruction of fatal events [100]. Inflammatory and immunohistochemical markers provide evidence of early ischemia and myocarditis when histology is negative, while systemic autoimmune and infectious diseases, including COVID-19, have further broadened the spectrum of forensic scenarios [103]. The most innovative contributions come from the field of molecular biomarkers. Cardiac microRNAs and exosomal RNAs have demonstrated stability and phenotype-specific patterns, while proteomic and metabolomic profiles reveal global metabolic signatures of ischemic collapse. The molecular autopsy, enabled by next-generation sequencing, has transformed the investigation of autopsy-negative deaths by identifying pathogenic variants in channelopathies and cardiomyopathies. This not only clarifies forensic diagnoses but also enables family cascade screening, bridging medico-legal and public health priorities [104]. Recent studies integrating multi-omics datasets with artificial intelligence have reached diagnostic accuracies exceeding 90%, pointing toward a new paradigm of data-driven, probabilistic forensic medicine [59].

Yet, despite these advances, major challenges remain. The absence of standardized cut-offs, variability in pre-analytical and post-analytical protocols, and limited multicenter validation continue to limit the routine application of biomarkers in forensic practice. Ethical and legal issues surrounding molecular autopsy, particularly regarding consent, privacy, and disclosure of genetic findings, also require urgent international consensus [105]. Without harmonization, even the most promising biomarkers risk being dismissed in court or inconsistently applied across jurisdictions. Looking forward, the integration of biomarkers into multimarker panels and multi-omics workflows, supported by artificial intelligence and standardized international guidelines, represents the most promising strategy to overcome current limitations. Collaborative research networks should prioritize large-scale validation studies and translational projects that bridge clinical and forensic cardiology. Only through this integrative, multidisciplinary approach will biomarkers achieve their full potential: not only clarifying the mechanisms of sudden cardiac death but also strengthening the reliability of forensic diagnoses, informing legal proceedings, and contributing to the prevention of future fatalities in families and society at large [106].

## Figures and Tables

**Table 1:** Main post-mortem biomarkers for sudden cardiac death: matrices, stability, advantages, and limitations.

Biomarker	Matrix	Post-Mortem Stability	Diagnostic Utility	Limitations
Troponins (cTnI, cTnT, hs-cTnT)	Femoral blood, pericardial fluid	Moderate (better in pericardial fluid, up to 48 h)	Gold standard for myocardial necrosis; high specificity for cardiomyocyte injury	Affected by hemolysis, PMI, resuscitation
CK-MB	Blood, pericardial fluid	Variable, degrades with PMI	Useful if combined with troponins; early rise	Skeletal muscle cross-reactivity, low specificity
H-FABP	Blood, pericardial fluid	Low (rapid degradation, <24 h)	Early marker of ischemia, rises before troponins	PMI-sensitive, less specific
GPBB	Blood	Limited validation	Early ischemia, energy metabolism marker	Not standardized, assay variability
NT-proBNP/BNP	Pericardial fluid, blood	Good stability (24–48 h)	Marker of heart failure, remodeling, hypertrophy	Influenced by renal failure, pulmonary HTN
Copeptin	Blood, vitreous humor	Excellent stability	Marker of acute stress, useful in ischemic SCD	Non-specific (sepsis, trauma also elevate)
sST2	Pericardial fluid	Emerging evidence	Marker of myocardial strain and fibrosis	Few forensic studies, no standard cut-off
Cytokines (IL-6, CRP, PTX-3)	Blood, CSF	Variable, influenced by bacterial translocation	Supportive for myocarditis, sepsis, inflammatory SCD	Non-specific, PMI-sensitive
IHC: Fibronectin, C5b-9, S100A1, Desmin	Myocardium (tissue)	High (tissue integrity dependent)	Detect early ischemia and vital reaction	Require expert pathologist, not quantitative
miRNAs (miR-1, miR-133, miR-208, miR-499)	Blood, tissue, exosomes	Excellent (stable > 48 h)	Differentiate ischemic vs. arrhythmic SCD	Assay standardization needed
Exosomal miRNAs	Plasma, pericardial fluid	Very high (protected from degradation)	Phenotype-specific profiles (ischemia vs. cardiomyopathy)	Limited forensic validation
NGS-based Molecular Autopsy	DNA (blood, tissue)	Stable indefinitely if preserved	Detects pathogenic variants in channelopathies and cardiomyopathies	High rate of VUS, ethical–legal issues

**Table 2:** Main Gene Categories Implicated in Sudden Unexplained Death.

Category	Representative Genes	Notes on Forensic Relevance
Channelopathies	*SCN5A*, *KCNQ1*, *KCNH2*, *RYR2*, *CACNA1C*, *CACNA1A*	Variants frequently identified in autopsy-negative SUD; CACNA1A recently implicated in arrhythmogenic vulnerability and sudden death [65].
Cardiomyopathies	*MYH7*, *MYBPC3*, *LMNA*, *DSP*, *PKP2*, *DSG2*, *DSC2*, *DES*	Genes commonly involved in hypertrophic, dilated, and arrhythmogenic cardiomyopathies; often present before structural changes are detectable at autopsy.
Mitochondrial/Metabolic Genes	*COX5B*, *TAZ*, *SDHA*	COX5B emerging as a biomarker of metabolic and mitochondrial dysfunction in unexplained sudden death [64].
Sarcomeric/Contractile Genes	*MYH6*, *ACTC1*, *TNNT2*	MYH6 recently proposed as a candidate gene for unexplained sudden death, associated with impaired contractile regulation [64].
Novel or Candidate Loci Identified in Recent Multi-Omics Studies	Multiple low-frequency variants from WES/WGS screens	Includes genes without previous annotation in cardiac pathology but recurrently identified in recent sequencing cohorts; require functional validation before clinical/forensic application.

**Table 3:** Pre-analytical and post-analytical factors influencing biomarker interpretation in sudden cardiac death.

Factor	Impact on Biomarker Levels	Implications for Forensic Practice
Sample site(Femoral vs. cardiac blood)	Central blood often shows artificially elevated troponins and CK-MB due to local leakage; femoral blood more reliable	Peripheral blood recommended for quantitative assays
Pericardial fluid	Protected from redistribution; stable for peptides and proteins (NT-proBNP, troponins)	Increasingly considered the gold matrix for cardiac biomarkers
Vitreous humor	Resistant to putrefaction; copeptin and electrolytes measurable; low hemolysis	Useful for stress biomarkers and metabolic markers when blood is degraded
CSF (cerebrospinal fluid)	Can contain cytokines, peptides, miRNAs; lower background contamination	Not yet standardized; limited forensic use
Post-mortem interval (PMI)	Longer PMI degrades small cytosolic proteins (H-FABP, GPBB) but troponins and NT-proBNP more stable	Interpretation should always be PMI-adjusted
Hemolysis	Raises CK-MB, LDH, AST; interferes with immunoassays	Visual inspection and exclusion of hemolyzed samples essential
Putrefaction/bacterial activity	Elevates cytokines (IL-6, CRP) and inflammatory markers; may mimic sepsis	Requires correlation with histology and culture
Sample storage	Improper freezing leads to RNA/protein degradation	Standard protocols: −80 °C for RNA/protein, DBS cards for DNA
Interpretive model	Single analyte cut-offs unreliable; ratios (pericardial fluid/blood) more robust	Bayesian and AI models integrating multi-marker panels are emerging

**Table 4:** Molecular and multi-omics biomarkers in post-mortem investigation of sudden cardiac death.

Approach/Biomarker	Matrix	Diagnostic Potential	Advantages	Limitations/Challenges
miRNAs (miR-1, miR-133, miR-208, miR-499)	Blood, myocardium, plasma	Differentiate ischemic SCD from arrhythmic deaths; correlate with troponins and histology	High tissue specificity; stable in post-mortem samples (up to 72 h)	Need for standardization of assays; variability between studies
Exosomal miRNAs	Plasma, pericardial fluid	Provide phenotype-specific signatures (ischemia, cardiomyopathy, arrhythmic SCD)	Protected from degradation; long-term stability; retrospective analysis feasible	Limited forensic validation; cost-intensive isolation methods
Proteomics	Myocardial tissue, blood	Identifies stress-response proteins (HSPs, annexins, mitochondrial enzymes)	Uncovers novel pathways of myocardial injury	Requires advanced instrumentation; not standardized for forensics
Metabolomics	Blood, pericardial fluid, vitreous	Characterizes energy failure (acylcarnitines, amino acids, TCA intermediates)	Reflects systemic collapse; PMI-adjusted models possible	Sensitive to PMI and storage; requires reference databases
Transcriptomics (mRNA, lncRNA)	Myocardial tissue	Detects ischemia-related gene expression (BNP mRNA, HIF-1α, miR-210)	Links molecular pathways with histology	Experimental; not yet in routine
NGS/Molecular Autopsy	DNA (blood, tissue)	Detects pathogenic variants in channelopathies (SCN5A, KCNQ1, RYR2) and cardiomyopathies (PKP2, DSP, MYH7)	Explains autopsy-negative SCD; enables cascade screening	High rate of VUS; ethical/legal concerns; cost
Multi-omics + AI	Integrated datasets (genomic, proteomic, metabolomic, transcriptomic)	>90% accuracy in classifying ischemic vs. non-ischemic SCD (machine learning models)	Probabilistic interpretation; handles complex data	Experimental; requires multicenter validation and legal acceptance

## Data Availability

No new data were created or analyzed in this study. Data sharing is not applicable to this article.
